# Using Geographic Momentary Assessment to Explore Spatial Environment Influences on Wellbeing in People With HIV

**DOI:** 10.1007/s10461-024-04527-4

**Published:** 2024-10-28

**Authors:** Rayna E. Gasik, Ethan A. Smith, Simone J. Skeen, Stephanie Tokarz, Gretchen Clum, Erica Felker-Kantor, Katherine P. Theall

**Affiliations:** 1 Department of Social, Behavioral, and Population Sciences, School of Public Health and Tropical Medicine, Tulane University, New Orleans, LA USA; 2 Department of Psychology, Hunter College, City University of New York, New York, NY USA; 3 Department of Epidemiology, School of Public Health and Tropical Medicine, Tulane University, New Orleans, LA USA; 4 Comprehensive Alcohol Research Center, School of Medicine, Louisiana State University Health Sciences Center, New Orleans, LA USA

**Keywords:** Geographic Ecological Momentary Assessment, Spatial Determinants of Health, HIV

## Abstract

Place-based socio-economic and racial inequities contribute to health disparities among people with HIV (PWH). We used geographic ecological momentary assessment (GEMA) methodologies to explore the interplay between psychosocial outcomes in daily diaries and community spatial measures among PWH in New Orleans. PWH (N = 69) were recruited from a clinic-based cohort study. Micro-longitudinal data were collected via smartphone-based daily diaries and geospatial tracking over 14 days. First, correlations were run between diary derived outcomes (e.g., feeling safe in current location, stress), and spatial measures (e.g., violent crime, alcohol outlets, and vacant lots) within a half-mile buffer around each diary point to understand the real time associations between mood and space. Next, we fit multilevel models, controlling for between-person effects, to assess within-person associations between accumulative spatial daily violence exposure (50m, 100m and 200m buffers), and measures of daily mood and coping. Violent crime, alcohol outlets and vacant lots were inversely correlated with feeling safe and positively correlated with perceived ease of obtaining drugs. Vacant lots were positively associated with stress and inversely correlated with positive mood. Within individuals, higher daily accumulated violence exposure was associated with increased rumination at the 50m buffer level, decreased trust in oneself and others at the 100m buffer, and decreased ratio of positive/negative mood at all buffers. Our results emphasize the intricate interplay between mental wellness, coping mechanisms, and spatial measures in PWH. Future research and interventions for PWH should consider how spatial factors may influence PWH in terms of mental health and care engagement.

## Introduction

Place is fundamental to health and wellbeing, particularly among people with HIV (PWH) [1–5]. Across the U.S., place-based, racially polarized inequities in socio-economic status (SES) are routinely linked to late HIV diagnosis [6, 7], antiretroviral therapy (ART) non-adherence, substance use and tenuous viral suppression, and disparate burdens of HIV-attributed mortality [1]. Studies in Georgia, Colorado, Missouri, and California have found that residence in Census tracts (CTs) with higher poverty, wealth inequality, and food insecurity is associated with decreased likelihood of viral suppression and ART uptake [8–11]. Furthermore, syndemic concentrations of poverty, substance misuse, and interpersonal violence (IPV) [12] are shown to worsen the burden of depression among cis women living with HIV across the U.S., while higher crime rates, fewer Christian institutions, and higher neighborhood-level density of alcohol outlets are associated with worse mental health in the American South [13, 14].

Racism in US policy has shaped neighborhood environments, leading to less favorable health conditions in neighborhoods that are economically disadvantaged and predominantly Black or Hispanic [15, 16]. For example, alcohol outlets are disproportionality concentrated in Black and low-income neighborhoods, despite similar or lower alcohol consumption in comparison to wealthier populations, a phenomenon tied to historical and current discrimination in local policy [17]. Proximity to alcohol outlets, particularly those that involve alcohol that can be taken offsite, has been associated with higher alcohol consumption [18] which in turn increases risk of alcohol related morbidity and interferes with progression along the HIV continuum of care [2, 19]. Vacant lots are more concentrated in neighborhoods that are lower income, predominantly Black, and have a higher HIV incidence [20, 21]. A systematic review of vacant lots and health found associations with stress, mental health, self-reported physical activity, and resting heart rate [22]. Other research has tied vacant homes to reduced neighborhood resources, such as access to healthy food [23].

The impacts of alcohol outlets and vacant lots on health can be at least partly attributed to their impact on violence. Research on interpersonal violence suggests that in approximately 63% of violence-related injuries, the victim, perpetrator, or both had recently consumed alcohol [24]. Some research suggests vacant lots may serve as hot spots for violence [21, 25]. A New Orleans study found that housing vacancies were significantly associated with neighborhood level property and violent crime [26]. Community violence itself represents another chronic environmental stressor, unraveling neighborhood social cohesion and, by extension, emotional and instrumental support networks [2, 26–28]. Older adult PWH in Alabama and across the rural American South describe loneliness and self-isolation, often attributed to fear of violent crime [28–30], apprehensions that have been echoed by PWH interviewed in New Orleans [32]. Violence victimization including IPV, law enforcement violence, and community assault are strongly linked to post-traumatic stress [33] and are predictive of maladaptive coping techniques such as hazardous alcohol and “hard” drug use [34, 35], upending progression along the HIV continuum of care [2, 26, 36].

Greater New Orleans, comprising Orleans and Jefferson Parish, exemplifies these interlocking, mutually compounding, place-based determinants of wellbeing among PWH. As of 2020 Greater New Orleans was home to 7,048 PWH (66.0% Black, 75.2% male) [37, 38]. With a murder rate 5.0 times the U.S. 2019 average [39], a poverty rate of 23.8%, and a $38,571 disparity between median Black versus median White household incomes in 2020 [40], Greater New Orleans represents a concentrated context of infrastructural deficits, physical disorder, and environmental stressors [41, 42]. Recent *New Orleans Alcohol Use in HIV* (NOAH) study findings describe the toll on New Orleanian PWH. Prevalence of adverse childhood experiences (ACEs) among NOAH-enrolled PWH was 3.5, more than twice the U.S. average of 1.6 [42], with accumulated ACEs linked to hazardous life course alcohol-use trajectories [43], and heavy-drinking dependent alcohol-use typologies evidencing relative increases in depression, PTSD, and anxiety symptoms, and ART non-adherence [35].

One limitation of place based and spatial studies of health in PWH is that they are reliant on CTs and similar static, administrative, operationalizations of “place” [2, 44]. In contrast, “activity spaces,” or the routine daily paths a person may travel [14], offer a more granular, high-fidelity depiction of within-person placed-based determinants of health behaviors [14, 44]. Research in the past decade has advanced methods, pairing continuously logged daily Global Positioning System (GPS) coordinates and repeated ecological momentary assessment (EMA) surveys, called geographic momentary assessment or GEMA [44–46]. This method has been used in substance use research to characterize craving and use patterns in reference to environmental availability of alcohol (i.e., alcohol outlets) and environmental disorder [48] and in mental health research to understand stress and mood triggers in the general population [49]. GEMA studies among PWH are, to date, less common [2, 47, 50].

Studies with PWH have typically either solely focused on EMA or included spatial tracking without an EMA component [47, 51]. One recent study using GEMA methods examined time spent at home and daily mood among PWH compared to HIV-seronegative adults, however spatial components were limited and did not include environmental measures commonly used in substance use and mental health research [52]. A 2020 systematic review of EMA methods in US HIV research noted that GEMA and geographic based EMA for PWH was lacking and warranted further research [47].

This research is part of a larger intervention with the ultimate goal of creating an mHealth mobile application to support coping in PWH [32]. As part of intervention development, PWH in New Orleans completed daily EMAs with continuous GPS tracking to better understand potential spatial triggers for this population. In the present analyses we examined the relationship between daily diary measures and community-level spatial exposures using GEMA data to: (1) explore the relationship between daily diary measures (mood and environmental perception) and real-time GPS spatial measures (neighborhood violence, alcohol outlets, and vacant lots) where daily diaries were taken; and (2) understand how accumulative daily violence exposure on individual PWH activity paths influences changes in daily mood and coping and to understand how prolonged exposure throughout the day may influence mood and mental health.

## Methods

### Study Design

This study was a micro-longitudinal GEMA study enrolling a subset of violence-exposed PWH from an ongoing parent study of in-care adults with HIV in New Orleans, Louisiana (*N* = 365). Details on the parent cohort study have been described elsewhere [53]. Exclusion criteria in the parent study included an acute illness within the last 6 weeks, acute intoxication, or pregnancy. Excluded patients were eligible for enrollment after resolution of criteria.

Participants for the current study were recruited from the parent study. The following eligibility criteria had to be met for participation: (1) provided consent in the parent study to be re-contacted for future research; (2) on antiretroviral therapy (ART) and (3) self-reported a personal history of violence or lived in a high-violence neighborhood at baseline in the parent study (*n* = 258). History of violence was defined as reporting childhood physical or sexual abuse, physical assault as an adult, endorsing moderate–high stress due to neighborhood crime, family, or relational violence. Living in a high-violence area was defined as living in a CT that had a violent crime rate at or above the 75th percentile for violent crime rates in tracts in Orleans Parish. Ethics approval was obtained from the Tulane University Biomedical Institutional Review Board (Ref# 860463).

Individuals who met eligibility criteria were contacted by phone and invited to participate in this study. Those who expressed interest were invited to an information and enrollment meeting at which eligibility criteria was confirmed and informed consent provided. Following enrollment, a baseline survey was administered, and participants were informed about the other data collection procedures which included electronic daily diaries and activity path tracking using a GPS smartphone application.

## Study Population

Of the initial 245 participants from the parent study who met inclusion criteria, 38 declined to participate in this study and 58 had phone numbers that were disconnected or incorrect. The remaining 149 were contacted and invited to participate in the current study. Three attempts were made to contact eligible participants. Of the 149 eligible participants, 89 enrolled. No significant differences were found in age, race, education, income level between those who enrolled and those who did not enroll.

## Data Collection

### Baseline

Baseline data were collected through an interviewer-administered survey at enrollment using REDCap [54, 55], and later merged with baseline data from the parent study [53].

## Daily Diary

Participants were sent smartphone-administered daily diaries three times a day (morning – 8:00 am, afternoon – 1:00 pm, and evening – 7:00 pm) for 14 days. Participants received a text message that contained an individualized link to the web-based survey administered by Qualtrics [56]. The surveys took approximately 5 min to complete. Participants were instructed to complete each survey within a three-hour window from when the text was sent. Surveys not completed within 30 min past each window were not included in the analysis.

### Geospatial Tracking

Geospatial data were collected over a 14-day period using a GPS tracking application developed by ActSoft Encore [57]. The application was installed and activated on participants’ smartphones, recording latitudinal and longitudinal coordinates every minute when moving and every hour when stationary.

## Measures

### Baseline

Baseline measures were used to obtain an understanding of the study population’s demographics and health history. The baseline measures of interest included demographic variables (i.e., age); adverse childhood experiences (ACEs); lifetime experience of intimate partner violence (IPV); anxiety, depression, and post-traumatic stress symptoms and alcohol consumption.

*ACEs* were measured using the Adverse Childhood Experiences Questionnaire from the Kaiser Permanente ACE study [58]. This questionnaire asked individuals about their exposure to various negative childhood experiences, such as psychological, physical, or sexual abuse, witnessing violence against their mother, and living with family members who suffered from substance abuse, mental illness, suicidal ideation, or incarceration before the age of 18. The total ACE score was calculated by adding up all the affirmative responses, which ranged from 0 to 10, with a higher score indicating more adverse experiences.

The Composite Abuse Scale assessed *lifetime experiences of IPV* [59]. This scale consisted of 12 items asking whether a participant had ever experienced forms of physical, sexual, verbal, or psychological abuse in an intimate partner relationship. These questions were summed and a score of one or more indicated at least one lifetime experience of IPV.

*Anxiety and depression* were measured using the Hospital Anxiety and Depression Scale (HADS), a validated and reliable screening tool consisting of 14 items (including 7-item subscales for anxiety and depression) [60].

*Post-traumatic Stress Disorder (PTSD)* was measured through the PTSD PCL-5, a 20 item self-report measure screening for PTSD-consistent symptoms in relation to an individual’s most traumatic life event [61]. A cutoff of 33 was considered borderline/clinically high and was used to operationalize acute post-traumatic stress symptomatology.

*Alcohol consumption* was measured through the Alcohol Use Disorders Identification Test brief screener (AUDIT-C) consisting of three questions covering frequency of drinking and binge drinking [62]. Questions were summed to receive a total possible range of 0–12. A score of eight or more was considered harmful or hazardous drinking.

### Daily Diary

Morning, afternoon, and evening daily diaries collected information on mood, coping, and location perception.

#### Location Perception Measures

Location based questions were drawn from previous work [63] and asked three times daily for each survey. Safety was measured through: “How safe do you feel in your current environment?” with response options: not at all, somewhat and very safe. Ease of alcohol access was assessed three times daily through “How easy would it be for you to get al.cohol if you wanted some where you are right now?” with response options, “not easy”, “somewhat easy”, “very easy.” Ease of drug access was assessed three times daily through “How easy would it be for you to get drugs if you wanted some where you are right now?” with response options, “not easy”, “somewhat easy”, “very easy.” Substance use in location was measured as: “How much drug use /alcohol use and/or drug dealing do you currently see in your location?” with response options: “none”, “some”, “a lot.”

#### Current Mood Measures

Stress was measured three times daily through a question “How much stress are you feeling right now?” with response options of “none”, “some”, or “a lot” asked three times a day. Measures of mood were assessed three times per day through a 14-item checklist modified from the Positive and Negative Affect Schedule (PANAS) scale (e.g., “Since your last diary entry/last night, how have you felt?”) [64]. Endorsed feelings were summed to create a positive mood summary (range 0–6) and negative mood summary (range 0–8). Then a ratio of positive mood to negative mood was created to assess if positive mood was reported more frequently even if both positive and negative moods were reported concurrently.

#### Daily Mental Health Measures

PTSD symptoms were measured twice daily through five questions on a five-point scale modified from the PTSD PCL-5 with a possible range of 0–25 [65]. Rumination was assessed twice per day through “I thought over and over again about my emotions” with options “none”, “some”, and “a lot.” Trust in oneself and others was measured using a summary score of five items coded 1 for yes and 0 for no from the Posttraumatic Maladaptive Beliefs Scale [66].

#### Coping Measures

Measures of coping were assessed once per day through a 14-item checklist asking whether participants had used each coping strategy that day. An average daily score was calculated for adaptive coping strategies and maladaptive coping strategies modified from Brief-COPE [67], and then a ratio measure of adaptive to maladaptive coping strategies was generated.

### Community-Level Spatial Exposures

Community-level exposures included alcohol availability, neighborhood violence and vacant and abandoned property. All exposures were available as point data with a latitude and longitude and were geocoded, plotted, and overlaid on the Louisiana census tract shapefile as well as GPS polyline files for each participant using Python (Python Software Foundation, Wilmington, DE) [68]. Point data on alcohol outlets were obtained from the 2021 North American Industry Classification System (NAICS), with measures calculated separately for onsite (e.g., bars) and offsite (e.g., liquor stores) alcohol outlets. Timestamped geocoded data on violent crime events (i.e., assault and homicide) were obtained from the New Orleans Police Department [69]. These data were downloaded monthly from March 2021 through October 2022 and linked to each participant’s 14-day activity path which were derived from the GPS points. Violent crime data were based on confirmed 911 calls and coded by crime type. Neighborhood vacant and cited property was obtained through a working contract between the City of New Orleans for research. Properties included those with a judgment by the City’s Code Enforcement Division for lots that were unoccupied (usually vacant with no structure) and that had one or more of the following violations: grass or vegetation growth higher than 18 inches tall; trash, debris, or evidence of illegal dumping; and/or growth of noxious vegetation, such as poison ivy.

### Data Analysis

Descriptive statistics were computed for baseline variables (demographic variables, history of violence and mental health), daily diaries and spatial measures. These included means and standard deviations for continuous variables, and frequencies and percentages for categorical variables. Next, we used correlation analyses to obtain a real time understanding of the relationship between selected daily diary measures (current mood and location perception) and community-level spatial exposures in the location where daily diaries were taken. In this analysis the unit was person-day-time as we were interested in examining the daily diary report of the participant on a specific day and time correlated with the GPS point location related variables on that same day and time. Time and date stamps were used to link daily diary entries with nearest available GPS datapoints. This was done by taking the time stamp (day/time) of each diary for each participant and matching it to available GPS coordinates closest to that time stamp within up to five hours prior (if data was unavailable coordinates were reported as missing). Point locations outside of Orleans Parish (~ 33%) were excluded from analyses given the availability of community-level exposure data was limited to Orleans Parish. Half mile buffers were created around each daily diary location point. Intersections of diary location points and yearly point data of violent crime, offsite alcohol outlets, onsite alcohol outlets, and cited lots were created to determine the number of points within each buffer. Spearman’s correlations with p values were then calculated between spatial measures and daily diary measures. Spearman’s correlations were used because current mood and location perception measures were ordinal, and Spearman’s correlation can be used between two variables that are ordinal, interval, or ratio [70]. Daily diary measures related to perception of current location and current mood were chosen given that they may be most likely influenced by the participant’s current location.

Finally, we aimed to understand the relationship between daily violent crime exposure along the activity path exposures and daily mood, coping and mental health within individuals. Two weeks (14 days) of activity paths were generated as described above. For this analysis we focused on violent crime, so activity path–based measures of exposure to violent crime were constructed as follows: First, 50-, 100-, and 200-meter buffers were created around the 14-day activity paths. Then, the spatial point data were plotted and aggregated within all polyline buffers along the activity path. Day averages were then calculated for both daily diary measures and for activity space violence exposures. Exposures were group mean centered (i.e., each individual’s 14-day exposure average was calculated and subtracted from their daily average) in order to obtain variance within individuals throughout the 14 days. Crude multilevel models with random intercepts, nesting daily diary entries within individuals, were run to test the associations between daily diary measures as outcomes and group mean centered activity space violence measures as exposures were run, with daily observations nested within individual participants [71]. We ran models uncontrolled and controlling for age, sex assigned at birth, education. However, we found no differences in results after controlling, so we presented the uncontrolled model.

## Results

Out of the 89 participants who enrolled and completed the baseline survey, 74 (83%) completed at least one daily diary and 10 (11%) completed all 42. There was a mean of 40 daily diaries completed per person with a standard deviation of 11.4 and a median of 36 diaries completed per person out of 42, the total expected. Seventy six% or 775 out of 1,022 expected morning diaries were completed, 73% or 746 of 1,022 expected afternoon diaries were completed, and 71% or 727 of 1,022 expected evening diaries were completed. Most diaries, 1,947, were completed on weekdays, representing 74% of expected completion while 301 were on weekends, representing 68% of expected completion. The GPS tracking recorded a total of 114,077 point locations, representing 15,452 h tracked across participants, with a mean of 200 h (SD = 87.84) and median of 223 per participant over 14 days. Average hours tracked per day was 14 (SD = 2.33) with a median of 15. Our analytic sample included *N* = 68 participants who had at least one diary completed and at least one hour of GPS data recorded.

Baseline and socio-demographic characteristics of study participants by completion of daily diaries are shown in Table 1. Participants were predominantly Black or African American (84%), had an income of less than 20,000 USD (86.5%) and had a mean age of 57 years. More than half of participants (56%) reported at least one lifetime instance of IPV. A significant portion of participants exhibited borderline/clinical levels of PTSD symptoms (27.0%), borderline/clinical anxiety (37.1%) and borderline/clinical depression (24.7%) at baseline. Those that were not in the analytic sample (either had not completed a daily diary or had no GPS data) were more frequently male (76.2%) compared to those in the analytic sample (60.3%) and all had an income of less than 20,000 USD. Additionally, those not in the sample tended to be older, more likely to have less than a high school education and less likely to have a college or higher education level.

**Table 1:** Baseline descriptive statistics

	Non-Analytic Sample	Analytic Sample	Overall
	(*N* = 21)	(*N* = 68)	(*N* = 89)
**Sex at Birth**			
Male	16 (76.2%)	41 (60.3%)	57 (64.0%)
Female	5 (23.8%)	27 (39.7%)	32 (36.0%)
**Race**			
Asian Pacific Islander	0 (0%)	1 (1.5%)	1 (1.1%)
Black/African American	18 (85.7%)	57 (83.8%)	75 (84.3%)
White	3 (14.3%)	10 (14.7%)	13 (14.6%)
**Age**			
Mean (SD)	62.9 (6.2)	56.7 (10.1)	58.1 (9.7)
Median [Min, Max]	63.0 [49.0, 72.0]	58.0 [28.0, 77.0]	60.0 [28.0, 77.0]
**Income**			
Less than $20,000	21 (100%)	56 (82.4%)	77 (86.5%)
$20,000 or more	0 (0%)	12 (17.6%)	12 (13.5%)
**Education**			
Less than High School	12 (57.1%)	19 (27.9%)	31 (34.8%)
High School/GED	7 (33.3%)	21 (30.9%)	28 (31.5%)
Some College or Higher	2 (9.5%)	28 (41.2%)	30 (33.7%)
**Intimate Partner Violence (IPV)**			
No Lifetime IPV Reported	9 (42.9%)	23 (33.8%)	32 (36.0%)
At least one IPV Reported in lifetime	9 (42.9%)	41 (60.3%)	50 (56.2%)
Missing	3 (14.3%)	4 (5.9%)	7 (7.9%)
**Adverse Childhood Experiences**			
Mean (SD)	3.6 (2.2)	3.00 (2.4)	3.17 (2.3)
Median [Min, Max]	3.3 [1.0, 8.0]	2.5 [0, 9.0]	2.5[0, 9.0]
Missing	3 (14.3%)	17 (25.0%)	20 (22.5%)
**Alcohol Use Disorders Identification Test Score (AUDIT)**			
Less than 8	15 (71.4%)	50 (73.5%)	65 (73.0%)
8 or More	6 (28.6%)	18 (26.5%)	24 (27.0%)
**Post Traumatic Stress Disorder (PTSD)**			
No/Low PTSD	17 (81.0%)	60 (88.2%)	77 (86.5%)
Borderline/Clinical PTSD	4 (19.0%)	8 (11.8%)	12 (13.5%)
**Anxiety**			
No/Low Anxiety	14 (66.7%)	42 (61.8%)	56 (62.9%)
Borderline/Clinical Anxiety	7 (33.3%)	26 (38.2%)	33 (37.1%)
**Depression**			
No/Low Depression	14 (66.7%)	53 (77.9%)	67 (75.3%)
Borderline/Clinical Depression	7 (33.3%)	15 (22.1%)	22 24.7%)

Note: Analytic Sample includes at least one daily diary and one hour of GPS data

Averages and ranges for daily diary and GPS generated measures are shown in Table 2. Daily diary measures and GPS measures were aggregated into mean responses on each day and are shown as the mean of the daily means of each variable. The mean PTSD symptom score was 1.83 (SD = 3.01) and the max score was 17 out of a possible 25, indicated that daily reporting among most people was not high, however there was a large variability among participants. Participants reported an average of 1.21 (SD = 0.44) out of 2 for daily levels of stress. Participants generally felt safe with a mean score of 1.77 (SD = 0.48) and a score of 2 representing the highest possible safety. The ease of accessing alcohol was moderately high with a mean score of 1.27 (SD = 0.83) with 2 representing the highest ease of accessing alcohol. Participants reported an average of 1.97 (SD = 1.75) positive emotions for positive mood while reporting of negative emotions was considerably lower; negative mood had a mean of 0.21 (SD = 0.57). The ratio of positive to negative mood was on average 1.08 (SD = 1.39), suggesting a balance skewed slightly towards a positive mood. Coping strategies varied, with maladaptive coping being less common, mean = 0.05 (SD = 0.13), compared to adaptive coping, mean = 0.15 (SD = 0.20). The ratio of adaptive to maladaptive coping was 1.12 (SD = 0.99), indicating a predominance of adaptive strategies. Environmental stressors within half-mile buffers around diary points included yearly violence counts (mean = 80.83; SD = 51.69), alcohol outlet counts (mean = 8.05; SD = 8.79, and reported vacant lots (mean = 179.58; SD = 135.05). Daily activity paths were cross referenced with violence data and compared to biweekly rates of violence for the week the daily diary was taken for violent crime. Daily exposure increased as buffer size increased for violent crime, ranging from an average of 0.04 (SD = 0.09) violent crime events per day for a 50-meter buffer along the activity path to 0.30 (SD = 0.51) violent crime events per day on the 200-meter buffer.

**Table 2:** Daily Diary and spatial measures descriptive statistics

Diary Variables (mean aggregated value by day)		Mean (SD)	Range
Current Stress		0.23 (0.44)	0–2
Feeling Safe		1.77 (0.48)	0–2
Ease of Accessing Alcohol		0.27 (0.83)	0–2
Ease of Accessing Drugs		0.93 (0.80)	0–2
Substance Use in Location		0.28 (0.53)	0–2
Positive Mood		1.97 (1.75)	0–6
Negative Mood		0.21 (0.57)	0–4
Ratio Positive: Negative Mood		1.08 (1.39)	0–5
Post Traumatic Stress Disorder		1.83 (3.01)	0–17
Maladaptive Coping		0.05 (0.13)	0-0.80
Adaptive Coping		0.15 (0.20)	0-0.90
Ratio Adaptive: Maladaptive Coping		1.12 (0.99)	0-3.9
Repetitive Thinking		0.64 (0.71)	0–2
Trust in Oneself and Others		3.06 (1.01)	0–4
**Half Mile Buffers Around Diary Points**		**Mean (SD)**	**Range**
Violent Crime Counts Yearly		80.83 (51.69)	0-700
Offsite Alcohol Outlets		9.11 (9.65)	0–68
Onsite Alcohol Outlets		53.43 (104.57)	0-306
Potential Vacant Lots		179.58 (135.05)	0-1617
**Daily Activity Paths (mean value aggregated by day)**		**Mean (SD)**	**Range**
Violent Crime Biweekly-50 m		0.04 (0.09)	0–1
Violent Crime Biweekly-100 m		0.12 (0.26)	0-1.63
Violent Crime Biweekly-200 m		0.30 (0.51)	0–4

**Fig. 1:**
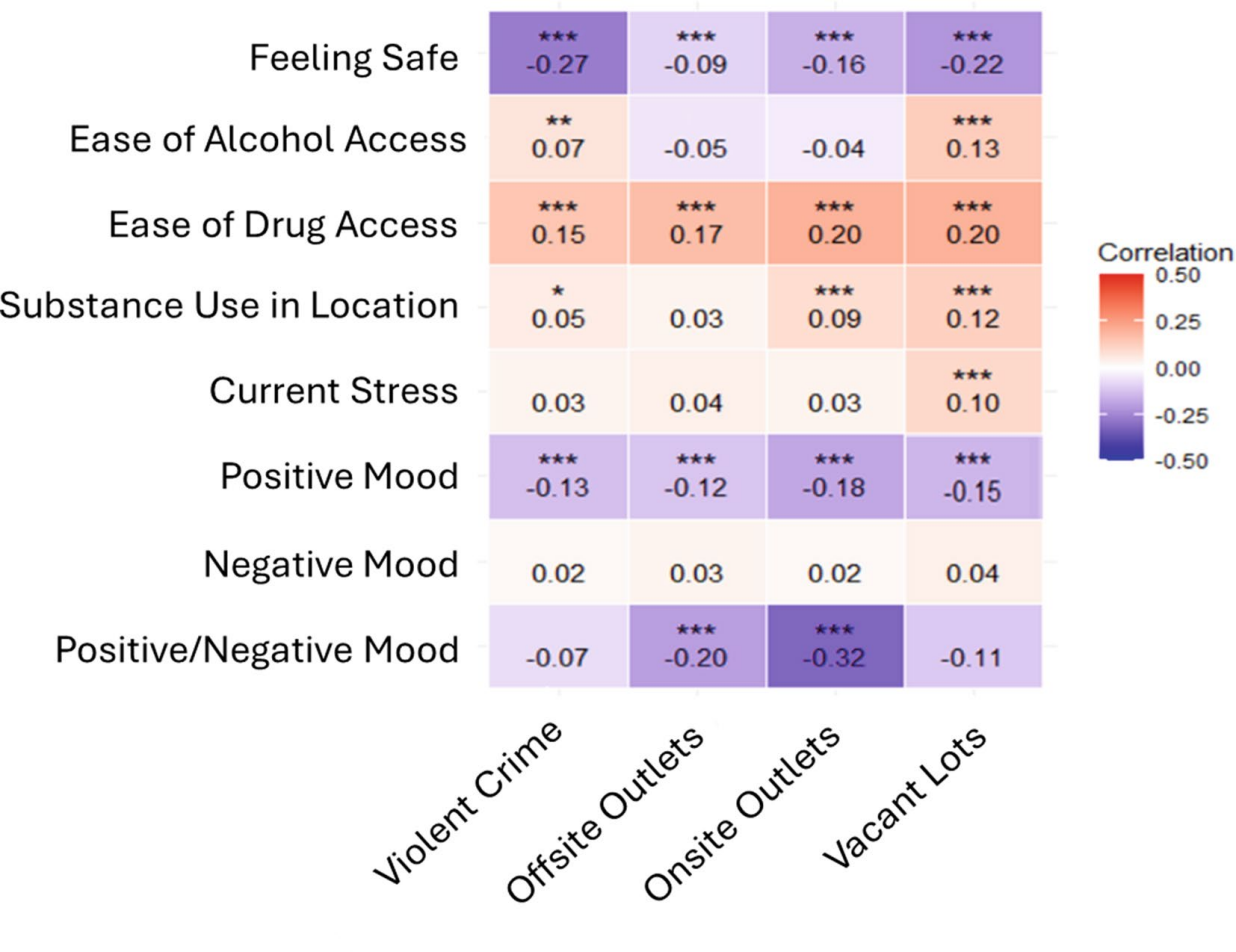
Correlation Between Daily Diary Perception of Location/Current Mood and Community Level Spatial Exposures at Day/Time of Diary Completion. *Note*: Spearman’s rank correlations were run on individual daily diaries (Y axis) and spatial measures (X axis) near where the daily diary was taken. *p < 0.05; **p < 0.01 ;***p < 0.001

A correlation matrix between daily diary variables and community-level spatial exposures around daily diary locations is displayed in Fig. 1. Feeling safe was significantly and negatively correlated with all community exposures, however correlation magnitudes were weaker for offsite alcohol (-0.09, *p* < 0.05) and onsite alcohol (-0.16, *p* < 0.001) and stronger for violent crime (-0.27, *p* < 0.0001) and vacant lots (-0.22, *p* < 0.0001). Ease of obtaining alcohol had a weak, significant, and positive relationship with vacant lots (0.13, *p* < 0.0001) and violent crime (0.07, *p* < 0.001). Ease of obtaining drugs similarly correlated with violent crime (0.15, *p* < 0.0001), offsite alcohol outlets (0.17, *p* < 0.0001), onsite alcohol outlets (0.20, *p* < 0.001), and vacant lots (0.20, *p* < 0.0001). Participant perceptions of how many individuals were using substances were positively and weakly correlated with vacant lots (0.12, *p* < 0.001), and onsite alcohol outlets (0.09, *p* < 0.0001). Vacant lots were significantly and positively correlated with current stress (0.10, *p* < 0.0001). All variables were significantly and negatively correlated with positive mood, with strengths ranging from –(0.12–0.18). The ratio of positive to negative mood was also correlated with offsite outlets (-0.20, *p* < 0.0001) and onsite alcohol outlets (-0.32, *p* < 0.0001).

**Fig. 2:**
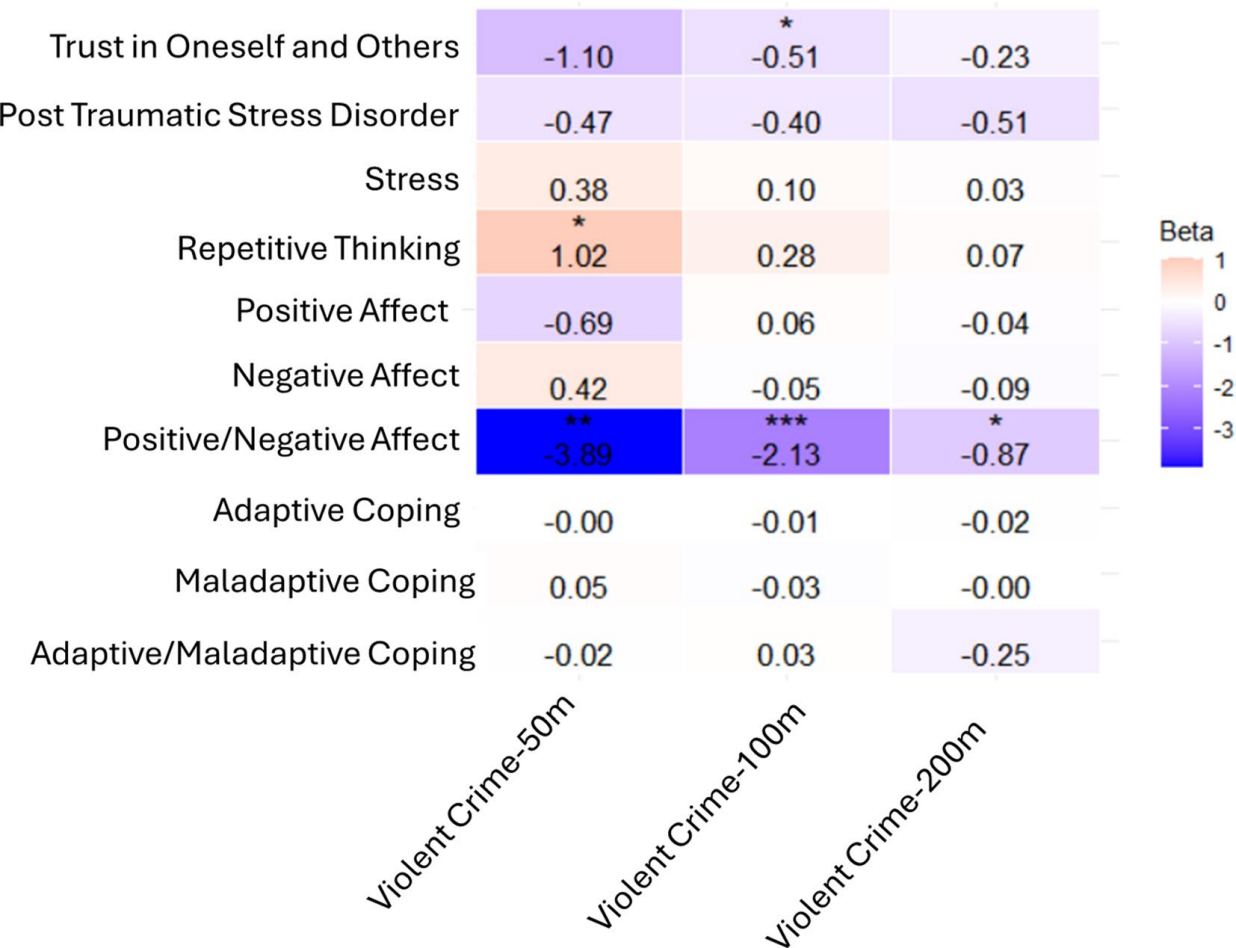
Regression Models of Spatial Exposures on the Daily Activity Path Predicting Mean Daily Mental Health Variables from Diaries. *Note: Multilevel linear regression models were run with mean daily diary entries (mean of 2–3 diary entries) as outcomes and group mean centered spatial variables as predictors.* *p < 0.05; **p < 0.01 ;***p < 0.001

The results of bivariate multilevel regressions with person-centered biweekly violent crime as the primary exposure and selected daily diary variables as outcomes are displayed in Fig. 2. Betas represent the impact of a one unit increase in violence from each person’s 14-day mean exposure. A negative association was observed between the ratio of positive mood to negative mood with violent crime. So, on days with higher-than-average activity space violence exposure, individuals reported significantly fewer positive emotions in relation to negative ones. The effect was greatest for violent crime within 50 m (B=-3.89, *p* < 0.001) and lower within 200 m (B=-0.87, *p* < 0.05). Though violent crime had a negative association with positive mood and a positive association with negative mood at the 50 m, this result was not significant and 100 m and 200 m levels had no associations with mood. Ruminative cognition was positively associated with violent crime; however, this association was only statistically significant for violent crime within 50 m (B = 1.02, *p* < 0.05). Trust in oneself and others was statistically significant and negatively associated with violent crime within 100 m (-0.51, *p* < 0.05). Coping strategies, including adaptive, maladaptive, and the ratio of adaptive to maladaptive coping and stress were not significantly related to violent crime exposure along the activity path Fig. 2.

## Discussion

In this study, we employed GEMA methodology to investigate the interplay between individuals’ daily emotional states and their interactions with the surrounding environment. We first examined community-level spatial measures for correlations with daily diary–reported moods and environmental perceptions in real time as the diaries were collected. We further explored the influence of violent crime events on daily mood and coping accumulatively across daily activity path. In our real time data analysis, potential vacant lots and violent crime were both correlated with perceptions of the environment (particularly feeling unsafe, and perceived availability of drugs) and participant’s current mood. In our activity path analysis, trust in oneself and others and mood were associated with violent crime events over PTSD and coping.

Our correlation analysis of real-time diary measures and spatial measures to the closest available location revealed several relationships between community-level spatial exposures and individual-level outcomes. Though correlations strengths were relatively weak (Spearman’s rho between 0.1 and 0.3), relationships between socio-emotional variables and place variables are likely multifactorial and complex. Violent crime was associated with feeling less safe and greater ease of obtaining alcohol or drugs. Previous research has suggested similar relationships between violent crime and both alcohol availability and perceived unsafety [72, 73]. Both violence and cited potential vacant lots were correlated with not feeling safe at similar strengths. Vacant lots was also the only spatial variable positively associated with current stress. This could be because violent crime and cited lots themselves were correlated spatially (0.37, *p* < 0.0001). However, participants’ perception of the connection between violent crime and vacant lots may be higher than these correlations. A study in Detroit found that feeling unsafe during daytime walks was associated with how many vacant or abandoned properties were located around a participant’s home [74]. A qualitative study in Philadelphia found that participants felt anxious and perceived threats to their physical safety when passing by areas with vacant lots [75]. This research is consistent with previous studies connecting neighborhood violent crime to fear of leaving the house and isolation in PWH [28–31]. Though the impact of vacant lots on feelings of safety has not been studied specifically in PWH, some research has suggested a correlation between concentration of vacant lots and incidence of HIV [20]. Additionally, proximity to care sites and transportation availability to care sites have been associated with viral suppression [76], suggesting the importance of PWH’s ability to feel safe while traveling within and outside of their neighborhoods to receive care.

In our analysis, perceived ease of obtaining drugs was positively associated with all selected spatial measures, particularly alcohol outlets and cited lots. Other research in Baltimore has suggested offsite alcohol outlets, but not onsite, were associated with rate of drug overdose in a neighborhood [77]. Though many studies have found a stronger association between offsite versus onsite alcohol outlets, a study in Philadelphia found that drug-related arrests were strongly and significantly spatially associated with both liquor stores (offsite) and establishments which sell beer (both onsite and offsite) [78]. Additionally, perception of how many people were using substances around a participant was positively associated with onsite but not offsite alcohol outlets, possibly because participants do not stay at offsite outlets for a prolonged period of time. This may also be related to unique ordinances around alcohol in the City of New Orleans, which allow individuals to move freely while carrying open alcoholic drinks [79]. Perception of ease of obtaining alcohol was not correlated with how many alcohol outlets were nearby but was correlated with potential vacant lots. This is surprising, as density of alcohol outlets has been related to alcohol consumption across multiple studies [80, 81]. However, a 2016 review argued that many of these studies are aggregated at the neighborhood level and other studies have found no association between alcohol outlets and consumption when controlling for individual-level factors [82]. It is also possible that participants considered ease of obtaining alcohol based on whether they already had alcohol at home (rather than purchasing) as studies indicate highest volume of alcohol consumption among adult drinkers is at home [83].

Our second analysis focused on the cumulative influence of activity path–based violent crime exposure on daily mood and coping. On days where violent crime exposure (measured biweekly) on the activity path was higher, participants reported reduced feelings of trust at the 100 m range, increased rumination about one’s emotions at the 50 m range, and fewer positive emotions in relation to negative ones for all ranges. Previous research has suggested that living in a neighborhood with higher crime is associated with reduced trust in one’s community and lower social capital [84]. However, this research does not distinguish between whether violence reduces ties or whether reduced ties lead to violence. Other research has suggested that individuals in neighborhoods with higher crime and less stability are less likely to have trust in health care systems [85, 86]. Trust in health care systems is vital to the wellbeing of PWH, with strong ties to engagement across the HIV continuum of care [87]. Violence was listed as a key barrier to medical care in a qualitative study of African American women with HIV in North Carolina, not only through generating distrust in the larger community but through feelings of unsafety while traveling to appointments [86]. Considering repetitive thought, or rumination, is an important factor in the development and maintenance of depression [88], our finding that rumination was tied to activity path exposure is consistent with previous literature. For example, a 2021 metanalysis of 63 studies found that neighborhood crime was significantly related to depression and psychological distress even when controlling for study characteristics, with the most robust studies indicating an 8–25% increased risk of poorer mental health in high crime areas [89]. This review did not report any studies using activity space as exposure, however this method was deemed valuable for future research. Given rumination and mood’s association with PTSD-consistent symptoms [90], we expected PTSD symptomatology to also be associated with violent crime. It is possible that participant’s reactions to higher exposure to violent crime areas was more emotion focused (lower positive moods in relation to negative mood and repetitive thinking about one’s emotions) rather than focused on their individual traumatic experience. Additionally, one study of post-traumatic growth found that intrusive thoughts and avoidant coping strategies were more predictive of growth than PTSD symptoms [91]. These results suggest that the interplay between PWH’s environment and emotional response is complex and warrants further study.

### Limitations

We acknowledge several limitations to this study. First, this is a preliminary analysis, and models we examined only controlled for age, sex at birth, and education. The sample size was small and the variability in mobility patterns (and some daily diary measures) was low. We began collecting data in 2020 during stay-at-home mandates of the Covid-19 pandemic, which likely resulted in reduced and altered mobility patterns compared to the pre-pandemic period and may have reduced variability in some participants. Additionally, in 2021, Hurricane Ida and its aftermath forced our team to temporarily suspend our study. Participants reported that 69.1% of their daily diaries were taken from home locations. This lack of variability reduced our ability to execute complex analyses from the data. Though many of the measures were in the expected direction, there were inconsistencies. For example, trust was only significantly and inversely associated with violent crime at the 100 m buffer size, even though the effect size was larger for the 50 m buffer. These inconsistencies could be due to a variety of factors: (1) Larger buffers include more data points and thus increase variability in the exposure. This could particularly explain why smaller buffers were not significant despite a larger effect size. (2) Spatial data can have inaccuracies in location of point data [92] and larger buffer sizes may better account for these errors. (3) There may be a threshold distance for certain exposures, where a neighborhood exposure’s influence on a participant is only apparent at more proximal levels. We also ran into a few issues considering the data quality of the participant’s GPS data. Data was meant to be collected every hour if the participant was stationary and every minute if they were moving. However, common to GEMA and similar studies we had some ‘GPS receiver error’ (gaps in GPS tracking) which resulted in some days not being used for analysis [93]. Though Qualtrics captured GPS points during diaries, there were considerable inaccuracies which meant we needed to rely on GPS tracking for the real time analysis.

## Conclusion

Our findings highlight the complex relationship between neighborhood stressors, mental health, and coping in PWH. Future research should consider the influence of personal experience and associations with neighborhood markers, such as vacant lots. For example, a study in Philadelphia used photovoice, interviews, and focus groups to create emotional maps of the neighborhood which categorize locations in the neighborhood (sites of dumping sites or disrepair, churches, community centers, etc.) with positive or negative emotions tied to personal histories [94]. Additionally using continuously measured biological data (such as heart rate and blood pressure) could aid in understanding the conscious and unconscious influence of neighborhood environment on an individual’s health. Finally, public health strategies must consider the broader environmental context in which PWH live and navigate daily. Experiencing neighborhood violence and disrepair is closely linked to concentrated disadvantage and structural inequalities, such as redlining, among PWH [15, 16]. The inequalities which PWH face, particularly Black and low-income PWH, also include exposure to neighborhood violence, transportation disparities, and food insecurity [11–13; 29, 30]. This research highlights the need for upstream approaches which address these structural disadvantages. Community-based participatory research serves as one promising approach which can aid in elucidating these the structural inequalities in individual communities and aid in policy decision making to better serve the needs of PWH [30].

## Data Availability

Not applicable.

## References

[1] Tieu HV, Koblin BA, Latkin C, et al. Neighborhood and network characteristics and the HIV care continuum among gay, bisexual, and other men who have sex with men. J Urban Health. 2020;97:592–608. 10.1007/s11524-018-0266-2. 29845586 10.1007/s11524-018-0266-2 PMC7560681

[2] Duncan DT, Kim B, Al-Aljouni YA, Callander D. Neighborhood-level structural factors, HIV, and communities of color. In: Ojikutu BO, Stone VE, edsSpring;. HIV in US Communities of Color. 2020:147-168.

[3] Remien RH, Stirratt MJ, Nguyen N, Robbins RN, Pala AN, Mellins CA. Mental health and HIV/AIDS: the need for an integrated response. AIDS. 2019;33:1411–20. 10.1097/QAD.0000000000002227. 30950883 10.1097/QAD.0000000000002227 PMC6635049

[4] Bowleg L, Malekzadeh AN, Mbaba M, Boone CA. Ending the HIV epidemic for all, not just some: structural racism as a fundamental but overlooked social-structural determinant of the US HIV epidemic. Curr Opin HIV AIDS. 2022;17:40–5. 10.1097/COH.0000000000000724. 35102051 10.1097/COH.0000000000000724 PMC9109814

[5] Pellowski JA, Kalichman SC, Matthews KA, Adler N. A pandemic of the poor: social disadvantage and the U.S. HIV epidemic. Am Psychol. 2013;68:197–209. 10.1037/a0032694. 23688088 10.1037/a0032694 PMC3700367

[6] Time to tackle late diagnosis. Lancet HIV. 2022;9:E139. 10.1016/S2352-3018(22)00040- 10.1016/S2352-3018(22)00040-6 35218733

[7] Ransome Y, Kawachi I, Braunstein S, Nash D. Structural inequalities drive late HIV diagnosis: the role of black racial concentration, income inequality, socioeconomic deprivation, and HIV testing. Health Place. 2016;42:148–58. 10.1016/j.healthplace.2016.09.004. 27770671 10.1016/j.healthplace.2016.09.004 PMC5584790

[8] Kalichman S, Shkembi B, Hernandez D, Katner H, Thorson KR. Income inequality, HIV stigma, and preventing HIV disease progression in rural communities. Prev Sci. 2019;20:1066–73. 10.1007/s11121-019-01013-5. 30955136 10.1007/s11121-019-01013-5 PMC7000177

[9] Vernon F, Morrow M, MaWhinney S, et al. Income inequality is associated with low cumulative antiretroviral adherence in persons with human immunodeficiency virus. Open Forum Infect Dis. 2020;7(10):ofaa391. 10.1093/ofid/ofaa391. 10.1093/ofid/ofaa391 PMC7539687 33072812

[10] Watson CW-M, Sundermann EE, Hussain MA, et al. Effects of trauma, economic hardship, and stress on neurocognition and everyday function in HIV. Health Psychol. 2019;38:33–42. 0.1037/hea0000688. 10.1037/hea0000688 PMC6309226 30372103

[11] Shacham E, Lian M, Önen NF, Donovan M, Overton ET. Are neighborhood conditions associated with HIV management? HIV Med., Perez-Brumer AG, Reisner SL. Poverty matters: Contextualizing the syndemic condition of psychological factors and newly diagnosed HIV infection in the United States. AIDS. 2014; 28:2763-2769. 10.1097/QAD.0000000000000491 10.1097/QAD.0000000000000491 PMC4243582 25418633

[12] Oldenburg CE, Perez-Brumer AG, Reisner SL. Poverty matters: Contextualizing the syndemic condition of psychological factors and newly diagnosed HIV infection in the United States. AIDS. 2014; 28:2763–2769. doi: 10.1097/QAD.0000000000000491 10.1097/QAD.0000000000000491 PMC4243582 25418633

[13] Wright IA, Reid R, Shahid N, et al. Neighborhood characteristics, intersectional discrimination, mental health, and HIV outcomes among black women living with HIV, Southeastern United States, 2019–2020. Am J Public Health. 2022;112:S433–43. 10.2105/AJPH.2021.306675. 35763751 10.2105/AJPH.2021.306675 PMC9241469

[14] Theall KP, Wallace M, Felker-Kantor E, et al. Neighborhood alcohol environment: differential effects on hazardous drinking and mental health by sex in persons living with HIV (PLWH). AIDS Behav. 2019;23(12):3237–46. 10.1007/s10461-019-02632-3. 31401740 10.1007/s10461-019-02632-3 PMC7467156

[15] Nightingale CH. Segregation: a global history of divided cities. University of Chicago Press; 2012.

[16] Steil JP, Kelly NF, Vale LJ, Woluchem MS. Furthering fair housing: Prospects for racial justice in America’s neighborhoods. Temple University; 2021.

[17] Trangenstein PJ, Gray C, Rossheim ME, Sadler R, Jernigan DH. Alcohol outlet clusters and population disparities. J Urb Health. 2020;97:123–36. 10.1007/s11524-019-00372-2 PMC7010879 31264024

[18] Sherk, A., Stockwell, T., Chikritzhs, T., Andréasson, S., Angus, C., Gripenberg, J.,… Woods, J. (2018). Alcohol consumption and the physical availability of take-away alcohol: systematic reviews and meta-analyses of the days and hours of sale and outlet density. *Journal of studies on alcohol and drugs*, *79*(1), 58-67. 29227232

[19] Bulsara SM, Wainberg ML, Newton-John TRO. Predictors of adult retention in HIV care: a systematic review. AIDS Behav. 2018;22:752–64. 10.1007/s10461-016-1644-y. 27990582 10.1007/s10461-016-1644-y PMC5476508

[20] Brawner BM, Guthrie B, Stevens R, Taylor L, Eberhart M, Schensul JJ. Place still matters: Racial/Ethnic and Geographic Disparities in HIV Transmission and Disease Burden. J Urban Health: Bull New York Acad Med. 2017;94(5):716–29. 10.1007/s11524-017-0198-2 PMC5610132 28879489

[21] South EC, Macdonald JM, Tam VW, Ridgeway G, Branas CC. Effect of abandoned housing interventions on gun violence, perceptions of safety, and substance use in black neighborhoods: a citywide cluster randomized trial. JAMA Intern Med. 2023;183(1):31–9. 10.1001/jamainternmed.2022.5460. 36469329 10.1001/jamainternmed.2022.5460 PMC9857286

[22] Sivak CJ, Pearson AL, Hurlburt P. Effects of vacant lots on human health: a systematic review of the evidence. Landsc Urban Plann. 2021;208:104020.

[23] Mui Y, Jones-Smith JC, Thornton RL, Pollack Porter K, Gittelsohn J. Relationships between vacant homes and food swamps: a longitudinal study of an urban food environment. Int J Environ Res Public Health. 2017;14(11):1426. 29160811 10.3390/ijerph14111426 PMC5708065

[24] Cherpitel CJ, Ye Y, Bond J, Room R, Borges G. Attribution of alcohol to violence-related injury: self and other’s drinking in the event. J Stud Alcohol Drugs. 2012;73(2):277–84. 10.15288/jsad.2012.73.277. 22333335 10.15288/jsad.2012.73.277 PMC3281985

[25] Sadatsafavi H, Sachs NA, Shepley MM, Kondo MC, Ruth A. Barankevich. Vacant lot remediation and firearm violence–a meta-analysis and benefit-to-cost evaluation. Landsc Urban Plann. 2022;218:104281. 10.1016/j.landurbplan.2021.104281.

[26] Chen X, Rafail P. Do housing vacancies induce more crime? A spatiotemporal regression analysis. Crime Delinquency. 2020;66(11):1579–605.

[27] Quinn K, Voisin DR, Bouris A, Schneider J. Psychological distress, drug use, sexual risks and medication adherence among young HIV-positive black men who have sex with men: exposure to community violence matters. AIDS Care. 2016;28:866–72. 10.1080/09540121.2016.1153596. 26917328 10.1080/09540121.2016.1153596 PMC4955550

[28] Quinn KG, Spector A, Takahashi L, Voisin DR. Conceptualizing the effects of continuous traumatic violence on HIV continuum of care outcomes for young Black men who have sex with men in the United States. AIDS Behav. 2021;25:758–72. 10.1007/s10461-020-03040-8. 32944841 10.1007/s10461-020-03040-8 PMC7886964

[29] Lee Y, Walton R, Jackson L, Batey DS. Community-level factors and HIV health among older people living with HIV (PLWH) in Alabama, United States: a qualitative analysis. J Assoc Nurses AIDS Care. 2021;32:589–98. 10.1097/JNC.0000000000000214. 33009174 10.1097/JNC.0000000000000214

[30] Jackson L, Lee Y, Batey DS. Structural violence within communities and its impact on the well-being of people with HIV (PWH). AIDS Care. 2023;35(2):265–70. 35727148 10.1080/09540121.2022.2088679

[31] Miles MS, Isler MR, Banks BB, Sengupta S, Corbie-Smith G. Silent endurance and profound loneliness: socioemotional suffering in African americans living with HIV in the rural South. Qual Health Res. 2011;21:489–501. 10.1177/1049732310387935. 21041516 10.1177/1049732310387935 PMC3073239

[32] Skeen SJ, Tokarz S, Gasik RE, et al. A trauma-informed, geospatially aware, just-in-time adaptive mHealth intervention to support effective coping skills among people living with HIV in New Orleans: development and protocol for a pilot randomized controlled trial. JMIR Res Protoc. 2023;12:e47151. 10.2196/47151. Published 2023 Oct 24. 37874637 10.2196/47151 PMC10630874

[33] Tang C, Goldsamt L, Meng J, et al. Global estimate of the prevalence of post-traumatic stress disorder among adults living with HIV: a systematic review and meta-analysis. BMJ Open. 2020;10:e032435. 10.1136/bmjopen-2019-032435. 32345695 10.1136/bmjopen-2019-032435 PMC7213849

[34] Bhatraju E, Liebschutz JM, Lodi S, et al. Post-traumatic stress disorder and risky opioid use among persons living with HIV and chronic pain. AIDS Care. 2021. 10.1080/09540121.2021.1876838. Advance online publication. 33535800 10.1080/09540121.2021.1876838 PMC8333265

[35] Madkour AS, Felker-Kantor E, Wallace M, et al. Latent alcohol use typologies and health status among a cohort of adults living with HIV. Alcohol Alcohol. 2019;54:584–92. 10.1093/alcalc/agz071. 31580404 10.1093/alcalc/agz071 PMC6895462

[36] Erickson M, Shannon K, Ranville F, et al. Interpersonal violence and other social-structural barriers associated with needing HIV treatment support for women living with HIV. J Interpers Violence. 2022;37:NP9926–52. 10.1177/0886260520983257. 33403922 10.1177/0886260520983257 PMC8507564

[37] AIDSVu. Local data: Orleans Parish, LA, Accessed. August 5, 2023. https://aidsvu.org/local- data/united-states/south/louisiana/orleans-parish/

[38] IAPAC. Fast-Track Cities Global Web Portal: New Orleans. Accessed August 5. 2023. https://www.fast-trackcities.org/resources/new-orleans

[39] Theall KP, Francois S, Bell CN, Anderson A, Chae D, LaVeist TA. Neighborhood police encounters, health, and violence in a southern city. Health Aff. 2022;41:228–36. 10.1377/hlthaff.2021.01428. 10.1377/hlthaff.2021.01428 PMC9037135 35130074

[40] Brink-Johnson A, Lubin J. Structural Racism in New Orleans: Facts, Figures & Opportunities for Advancing Racial Equity. Center for Urban and Racial Equity; 2020. Accessed August 5, 2023. https://urbanandracialequity.org/wp-content/uploads/2020/08/Structural-Racism- in-New-Orleans.pdf

[41] Bailey RK, Barker CH, Grover A. Structural barriers associated with the intersection of traumatic stress and gun violence: a case example of New Orleans. Healthcare. 2021;9:1645. 10.3390/healthcare9121645. 34946370 10.3390/healthcare9121645 PMC8701294

[42] Wallace M, Felker-Kantor E, Madkour A, et al. Adverse childhood experiences, smoking and alcohol use, and allostatic load among people living with HIV. AIDS Behav. 2020;24:1653–62. 10.1007/s10461-019-02684-5. 31559525 10.1007/s10461-019-02684-5 PMC7096273

[43] Madkour AS, Clum GA et al. Childhood hardship and health status among persons living with HIV (PLWH): do lifetime alcohol use trajectories mediate? 2022 APHA Annual Meeting and Expo. November 2022;6-9. Boston, MA. https://apha.confex.com/apha/2022/meetingapi.cgi/Paper/518734?filename=2022_Abstract518734.pdf&template=Word

[44] Campanella R. City neighborhoods: a matter of evolving perception. The Lens. Published June 1, 2011. Accessed November 6, 2022. https://thelensnola.org/2011/06/01/new-orleans- neighborhoods-the-73-census-tracts-creole-treme-marigny-sixth-ward/

[45] Shacham E, Scroggins SE, Ellis M. Implementing geospatial science and technology to get to zero new HIV infections. Curr HIV/AIDS Rep. 2023;20:139–47. 10.1007/s11904-023-00658-w. 37145264 10.1007/s11904-023-00658-w

[46] Theall KP, Felker-Kantor E, Wallace M, Zhang X, Morrison CN, Wiebe DJ. Considering high alcohol and violence neighborhood context using daily diaries and GPS: a pilot study among people living with HIV. Drug Alcohol Depend. 2018;187:236–41. 10.1016/j.drugalcdep.2018.03.005. 29684891 10.1016/j.drugalcdep.2018.03.005 PMC5959796

[47] Smiley SL, Milburn NG, Nyhan K, Taggart T. A systematic review of recent methodological approaches for using ecological momentary assessment to examine outcomes in U.S. based HIV research. Curr HIV/AIDS Rep. 2020;17:333–42. 10.1007/s11904-020-00507-0. 32594365 10.1007/s11904-020-00507-0 PMC11230647

[48] Freisthler B, Lipperman-Kreda S, Bersamin M, Gruenewald PJ. Tracking the when, where, and with whom of alcohol use: integrating ecological momentary assessment and geospatial data to examine risk for alcohol-related problems. Alcohol Research: Curr Reviews. 2014;36(1):29. 10.35946/arcr.v36.1.04 PMC4432856 26258998

[49] Kou L, Tao Y, Kwan MP, Chai Y. Understanding the relationships among individual-based momentary measured noise, perceived noise, and psychological stress: a geographic ecological momentary assessment (GEMA) approach. Health Place. 2020;64:102285. 32819555 10.1016/j.healthplace.2020.102285

[50] Rodriguez-Diaz CE, Davis W, Ellis MV, et al. Disrupting the systems: opportunities to enhance methodological approaches to address socio-structural determinants of HIV and end the epidemic through effective community engagement. AIDS Behav. 2021;25:225–31. 34618266 10.1007/s10461-021-03475-7 PMC8494756

[51] Duncan DT, Regan SD, Park SH, et al. Assessment of spatial mobility among young men who have sex with men within and across high HIV prevalence neighborhoods in New York City: the P18 neighborhood study. Spat Spatiotemporal Epidemiol. 2020;35:100356. 10.1016/j.sste.2020.100356. 33138958 10.1016/j.sste.2020.100356 PMC7609976

[52] Kamalyan, L., Yang, J. A., Pope, C. N., Paolillo, E. W., Campbell, L. M., Tang, B.,… Moore, R. C. (2021). Increased social interactions reduce the association between constricted life-space and lower daily happiness in older adults with and without HIV: a GPS and ecological momentary assessment study. *The American Journal of Geriatric Psychiatry*, *29*(8), 867-879. 10.1016/j.jagp.2020.11.005 PMC8134622 33293248

[53] Welsh DA, Ferguson T, Theall KP, Simon L, Amedee A, Siggins RW, Nelson S, Brashear M, Mercante D, Molina PE. The New Orleans alcohol use in HIV study: launching a translational investigation of the interaction of alcohol use with biological and socioenvironmental risk factors for multimorbidity in people living with HIV. Alcoholism: Clin Experimental Res. 2019;43(4):704–9. 10.1111/acer.13980 PMC6443470 30748025

[54] Harris PA, Taylor R, Minor BL, et al. The REDCap consortium: building an international community of software platform partners. J Biomed Inf. 2019;95:103208. 10.1016/j.jbi.2019.103208. 10.1016/j.jbi.2019.103208 PMC7254481 31078660

[55] Harris PA, Taylor R, Thielke R, Payne J, Gonzalez N, Conde JG. Research electronic data capture (REDCap)—a metadata-driven methodology and workflow process for providing translational research informatics support. J Biomed Inf. 2009;42:377–81. 10.1016/j.jbi.2008.08.010. 10.1016/j.jbi.2008.08.010 PMC2700030 18929686

[56] Miller CA, Guidry JP, Dahman B, Thomson MD. A tale of two diverse qualtrics samples: information for online survey researchers. Cancer Epidemiol Biomarkers Prev. 2020;29(4):731–5. 10.1158/1055-9965.EPI-19-0846. 32066616 10.1158/1055-9965.EPI-19-0846 PMC8758054

[57] Encore homepage. Encore. URL: https://workforce.actsoft.com/encore/ [accessed 2023-01-05].

[58] Felitti VJ, Anda RF, Nordenberg D, Williamson DF, Spitz AM, Edwards V, Marks JS. Relationship of childhood abuse and household dysfunction to many of the leading causes of death in adults: the adverse childhood experiences (ACE) study. Am J Prev Med. 1998;14(4):245–58. 9635069 10.1016/s0749-3797(98)00017-8

[59] Ford-Gilboe M, Wathen CN, Varcoe C, et al. Development of a brief measure of intimate partner violence experiences: the composite abuse scale (Revised)-Short form (CASR-SF). BMJ Open. 2016;6:e012824. 10.1136/bmjopen-2016-012824. 27927659 10.1136/bmjopen-2016-012824 PMC5168640

[60] Zigmond AS, Snaith RP. The hospital anxiety and depression scale. Acta Psychiatr Scand. 1983;67:361–70. 10.1111/j.1600-0447.1983.tb09716. 6880820 10.1111/j.1600-0447.1983.tb09716.x

[61] Blevins CA, Weathers FW, Davis MT, Witte TK, Domino JL. The posttraumatic stress disorder checklist for DSM-5 (PCL-5): development and initial psychometric evaluation. J Trauma Stress. 2015;28:489–98. 10.1002/jts.22059. 26606250 10.1002/jts.22059

[62] Bush K, Kivlahan DR, McDonell MB, Fihn SD, Bradley KA. The AUDIT alcohol consumption questions (AUDIT-C): an effective brief screening test for problem drinking. Ambulatory Care Quality Improvement Project (ACQUIP). Alcohol Use disorders Identification Test. Arch Intern Med. 1998;158:1789–95. 10.1001/archinte.158.16.1789. 9738608 10.1001/archinte.158.16.1789

[63] Elo IT, Mykyta L, Margolis R, Culhane JF. Perceptions of neighborhood disorder: the role of individual and neighborhood characteristics. Soc Sci Q. 2009;90(5):1298–320. 10.1111/j.1540-6237.2009.00657.x. 20174462 10.1111/j.1540-6237.2009.00657.x PMC2822409

[64] Watson D, Clark LA, Tellegen A. Development and validation of brief measures of positive and negative affect: the PANAS scales. J Personal Soc Psychol. 1988;54(6):1063. 10.1037//0022-3514.54.6.1063 3397865

[65] Erwin MC, Dennis PA, Coughlin LN, Calhoun PS, Beckham JC. Examining the relationship between negative affect and posttraumatic stress disorder symptoms among smokers using ecological momentary assessment. J Affect Disord. 2019;253:285–91. 31077971 10.1016/j.jad.2019.04.035 PMC6620145

[66] Vogt DS, Shipherd JC, Resick PA. Posttraumatic Maladaptive beliefs Scale: evolution of the personal beliefs and reactions Scale. Assessment. 2012;19:308–17. 10.1177/1073191110376161. 20660470 10.1177/1073191110376161

[67] Solberg MA, Gridley MK, Peters RM. The factor structure of the brief cope: a systematic review. West J Nurs Res. 2022;44(6):612–27. 33942676 10.1177/01939459211012044

[68] Van Python G, Drake FL. Python 3 reference Manual. Scotts Valley, CA: CreateSpace; 2009.

[69] City of New Orleans. Data Driven NOLA [Internet]. New Orleans: City of New Orleans; c2023 [cited 2024 Apr 19]. https://datadriven.nola.gov/home/

[70] Sprent P. and Nigel C. Smeeton. Applied Nonparametric Statistical methods. 4th ed. Boca Raton, FL: Chapman & Hall/CRC; 2007.

[71] Luke DA. Multilevel modeling. SAGE; 2020.

[72] Camacho Doyle M, Gerell M, Andershed H. Perceived Unsafety and Fear of Crime: the Role of Violent and Property Crime, Neighborhood characteristics, and prior Perceived Unsafety and Fear of Crime. Deviant Behav. 2022;43(11):1347–65. 10.1080/01639625.2021.1982657.

[73] Lardier DT, Reid RJ, Yu D, Garcia-Reid P. A Spatial Analysis of Alcohol Outlet Density and Abandoned properties on violent crime in Paterson New Jersey. J Community Health. 2020;45(3):534–41. 10.1007/s10900-019-00772-0. 31691088 10.1007/s10900-019-00772-0

[74] Pearson AL, Clevenger KA, Horton TH, Gardiner JC, Asana V, Dougherty BV, Pfeiffer KA. Feelings of safety during daytime walking: associations with mental health, physical activity and cardiometabolic health in high vacancy, low-income neighborhoods in Detroit. Mich Int J Health Geographics. 2021;20(1):1–13. 10.1186/s12942-021-00271-3 PMC8091672 33941196

[75] Garvin E, Branas C, Keddem S, Sellman J, Cannuscio C. More than just an eyesore: local insights and solutions on vacant land and urban health. J Urban Health. 2013;90(3):412–26. 10.1007/s11524-012-9782-7. Epub 2012/11/29. 23188553 10.1007/s11524-012-9782-7 PMC3665973

[76] Kahana SY, Jenkins RA, Bruce D, Fernandez MI, Hightow-Weidman LB, Bauermeister JA. Structural determinants of antiretroviral therapy use, HIV care attendance, and viral suppression among adolescents and young adults living with HIV. PLoS ONE. 2016;11(4):e0151106. 10.1371/journal.pone.0151106. & Adolescent Medicine Trials Network for HIV/AIDS Interventions 10.1371/journal.pone.0151106 PMC4817971 27035905

[77] Nesoff ED, Milam AJ, Morrison C, Weir BW, Branas CC, Furr-Holden DM, Martins SS. Alcohol outlets, drug paraphernalia sales, and neighborhood drug overdose. Int J Drug Policy. 2021;95:103289. 33984684 10.1016/j.drugpo.2021.103289 PMC8530831

[78] McCord ES, Ratcliffe JH. A micro-spatial analysis of the demographic and criminogenic environment of drug markets in Philadelphia. Australian New Z J Criminol. 2007;40(1):43–63.

[79] Ehrenfeucht R, Croegaert A. Learning from New Orleans: will revising or relaxing Public Space Ordinances Create a Just Environment for Street Commerce?.

[80] Gruenewald PJ. Regulating availability: how access to alcohol affects drinking and problems in youth and adults. Alcohol Res Health. 2011;34(2):248. 22330225 PMC3860569

[81] Campbell CA, Hahn RA, Elder R. The effectiveness of limiting alcohol outlet density as a means of reducing excessive alcohol consumption and alcohol-related harms. Am J Prev Med. 2009;37:556–69. 19944925 10.1016/j.amepre.2009.09.028

[82] Gmel G, Holmes J, Studer J. Are alcohol outlet densities strongly associated with alcohol-related outcomes? A critical review of recent evidence. Drug Alcohol Rev. 2016;35(1):40–54. 26120778 10.1111/dar.12304

[83] Sumetsky N, Gruenewald PJ, Lipperman-Kreda S, Lee JP, Mair C. Alcohol use frequencies and associated problems across drinking contexts. J Stud Alcohol Drug. 2022;83(1):91–8. 10.15288/jsad.2022.83.91 PMC8819891 35040764

[84] Garcia RM, Taylor RB, Lawton BA. Impacts of violent crime and neighborhood structure on trusting your neighbors. Justice Q. 2007;24(4):679–704.

[85] Yang TC, Matthews SA, Shoff C. Individual health care system distrust and neighborhood social environment: how are they jointly associated with self-rated health? J Urb Health. 2011;88:945–58. 10.1007/s11524-011-9561-x PMC3191206 21455831

[86] *Health Education & Behavior*, *49*(6), 1022-1032. 10.1177/10901981221109138 PMC9574897 35856333

[87] He N, Cleland CM, Gwadz M, Sherpa D, Ritchie AS, Martinez BY, Collins LM. Understanding medical distrust among African American/Black and latino persons living with HIV with sub-optimal engagement along the HIV care continuum: a machine learning approach. SAGE open. 2021;11(4):21582440211061314. 10.1177/21582440211061314 PMC9262282 35813871

[88] Cano-López JB, Garcia-Sancho E, Fernández-Castilla B, Salguero JM. Empirical evidence of the metacognitive model of rumination and depression in clinical and nonclinical samples: a systematic review and meta-analysis. Cogn Therapy Res. 2022 Apr;1:1–26.

[89] Baranyi G, Di Marco MH, Russ TC, Dibben C, Pearce J. The impact of neighbourhood crime on mental health: a systematic review and meta-analysis. Soc Sci Med. 2021;282:114106. 34139480 10.1016/j.socscimed.2021.114106

[90] Moulds ML, Bisby MA, Wild J, Bryant RA. Rumination in posttraumatic stress disorder: a systematic review. Clin Psychol Rev. 2020;82:101910. 10.1016/j.cpr.2020.101910. 32971312 10.1016/j.cpr.2020.101910

[91] Brooks M, Graham-Kevan N, Robinson S, Lowe M. Trauma characteristics and posttraumatic growth: the mediating role of avoidance coping, intrusive thoughts, and social support. Psychol Trauma: Theory Res Pract Policy. 2019;11(2):232. 10.1037/tra0000372 29723030

[92] Kirchner TR, Shiffman S. Spatio-temporal determinants of mental health and well-being: advances in geographically-explicit ecological momentary assessment (GEMA). Soc Psychiatry Psychiatr Epidemiol. 2016;51:1211–23. 27558710 10.1007/s00127-016-1277-5 PMC5025488

[93] Mennis J, Mason M, Ambrus A, Way T, Henry K. The spatial accuracy of geographic ecological momentary assessment (GEMA): error and bias due to subject and environmental characteristics. Drug Alcohol Depend. 2017;178:188–93. 28654871 10.1016/j.drugalcdep.2017.05.019

[94] Meenar MR, Mandarano LA. Using photovoice and emotional maps to understand transitional urban neighborhoods. Cities. 2021;118:103353. 10.1016/j.cities.2021.103353.

